# Low apolipoprotein M serum levels correlate with Systemic lupus erythematosus disease activity and apolipoprotein M gene polymorphisms with Lupus

**DOI:** 10.1186/s12944-017-0476-8

**Published:** 2017-05-05

**Authors:** Wenhan Du, Ting Shen, Hui Li, Yinyin Liu, Lagu He, Li Tan, Min Hu, Yaping Ren

**Affiliations:** Department of Laboratory Medicine, The Second Xiangya Hospital, Central South University, Changsha, Hunan 410011 China

**Keywords:** *APOM*, Systemic lupus erythematosus, Genetic polymorphism, Risk factor

## Abstract

**Background:**

Sequence variation in gene promoters is often associated with disease risk. In this study, we tested the hypothesis that common promoter variation in the *APOM* gene is associated with systemic lupus erythematosus (SLE) risk and SLE-related clinical phenotypes in a Chinese cohort. Meanwhile, we investigated the expression of apolipoprotein M (APOM) in the serum of patients with systemic lupus erythematosus (SLE) and its relationship with disease activity.

**Methods:**

We used a case-control design and genotyped 52 SLE patients and 52 healthy controls for 19 *APOM* promoter single nucleotide polymorphism (SNP) (rs113947529, rs1143030, rs114826514, rs116715239, rs12525463, rs1266078, rs2273612, rs28432254, rs34490746, rs4947251, rs55880811, rs707921, rs74890500, rs75629491, rs76611345, rs76794541, rs805264, rs805297, rs9267528). Genotyping was done by matrix-assisted laser desorption/ionization time-of-flight mass spectrometry (MALDI-TOF MS). The blood serum concentration of APOM was measured by an enzyme-linked immunosorbent assay in SLE patients and controls.

**Results:**

The average concentration of APOM in serum was significantly lower in SLE patients compared to controls and APOM levels in SLE patients with positive anti-dsDNA antibodies were dramatically lower than that of patients with negative anti-dsDNA antibodies (*P* = 0.011). It was interesting that APOM levels correlated with systemic lupus erythematosus disease activity index (SLEDAI) scores (*r* = −0.396, *P* = 0.004). No association between *APOM* and SLE susceptibility was detected in our Han Chinese cohort.

**Conclusions:**

Our results demonstrated that lower APOM levels in SLE patients and correlated with disease activity.

**Electronic supplementary material:**

The online version of this article (doi:10.1186/s12944-017-0476-8) contains supplementary material, which is available to authorized users.

## Background

Systemic lupus erythematosus (SLE) is a prototypic autoimmune inflammatory disease with a strong genetic component. The estimated prevalence is 37.7/100,000 in China [1]. Females are more commonly affected than males, with reported ratios of 9.2:1 [2]. Researches [3–5] demonstrate that major histocompatibility complex (MHC) polymorphisms, including single nucleotide polymmorphisms (SNPs) and classical human leukocyte antigen (HLA) alleles, have consistently been observed to be associated with SLE. Although increasing amounts of SNPs contribute to the risk of SLE in MHC region were found, there are still new SNPs need to be detected.

Apolipoprotein M (APOM) is a 26-kDa newly identified human apolipoprotein, mainly expressed in the liver and kidney [6]. *APOM* spans 2.3 kilobases (kb) on chromosome 6p21.3 within the major histocompatibility complex class III (MHC III) region and is comprised of 6 exons and 5 introns, that may play a crucial role in the immune response. It has been reported that *APOM* gene SNP confer the risk of rheumatoid arthritis (RA) [7, 8], type1 diabetes (T1D) [9], T2D [10, 11], coronary artery disease (CAD) [12] and ischemic stroke [13]. However, the association of genetic variation in the promoter region of *APOM* with SLE risk has not been reported yet. The aim of the current study is to discover the relationship between serum APOM levels and SLE patients, and identify SNPs in the promoter region of *APOM* could be a causative genetic factor in the risk for developing SLE in a Chinese cohort.

## Methods

### Subjects

All patients fulfilled the criteria for SLE set by the America College of Rheumatology (ACR) classification in 1982 [14]. The patients were recruited from the Second Xiangya hospital between August 2014 and October 2015. From the 128 individuals screened, 52 newly diagnosed SLE patients were enrolled in this study. The exclusion criteria for SLE were as fellows: (1) receiving any steroid or immunosuppressant treatments before blood samples were collected; (2) accompanied with liver or kidney disease. In addition, of the 120 controls screened for participation, 21 refused to participate, 27 failed the eligibility criteria and 20 were excluded due to the consistence of sex ratio with SLE patients. Hence, 52 controls were eventually evaluated in this study (Additional file 1: Figure S1). The volunteers underwent a physical examination in our hospital. The study protocol was reviewed and approved by The Second Xiangya Hospital Investigational Review Board. Informed consent was obtained from all participants.

### SNP selection and genotyping analysis

**Fig. 1:**
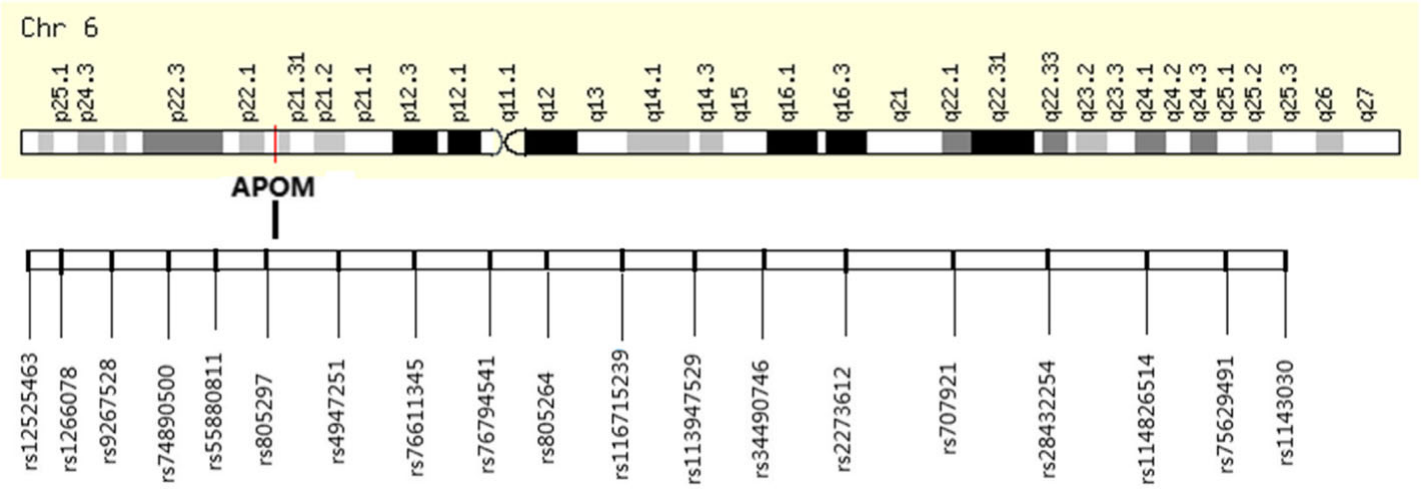
Genetic map of used SNPs for the *APOM* promoter region

Blood samples were collected and transferred to test tubes containing ethylenediamine tetra-acetic acid (EDTA). Genomic DNA was isolated from whole blood using the DNA Blood Kit (Daan, Guangzhou, China). Nineteen APOM SNPs including rs113947529, rs1143030, rs114826514, rs116715239, rs12525463, rs1266078, rs2273612, rs28432254, rs34490746, rs4947251, rs55880811, rs707921, rs74890500, rs75629491, rs76611345, rs76794541, rs805264, rs805297, rs9267528 enlisted in the NCBI database (Table 1) were selected for genotyping by MALDI-TOF MS using the Mass ARRAY system (Sequenom, San Diego, CA, USA) at the Beijing Genomics Institute (Shenzhen, China) (Fig. 1). The overall call rate was 95.3%. For quality control, repeated analyses were undertaken on 10% of randomly selected samples.

**Table 1:** Available *APOM* SNPs in proximal promoter region (http://www.ncbi.nlm.nih.gov/SNP).

	SNP	Chromosome	Region
1	rs12525463	6:31,654,007	Promoter
2	rs1266078	6:31,654,266	Promoter
3	rs9267528	6:31,654,364	Promoter
4	rs74890500	6:31,654,571	Promoter
5	rs55880811	6:31,654,754	Promoter
6	rs805297	6:31,654,829	Promoter
7	rs4947251	6:31,654,885	Promoter
8	rs76611345	6:31,655,297	Promoter
9	rs76794541	6:31,655,639	Promoter
10	rs805264	6:31,656,096	Promoter
11	rs116715239	6:31,656,393	Promoter
12	rs113947529	6:31,656,688	Promoter
13	rs34490746	6:31,657,231	Promoter
14	rs2273612	6:31,657,557	Promoter
15	rs707921	6:31,657,764	Promoter
16	rs28432254	6:31,658,107	Promoter
17	rs114826514	6:31,658,209	Promoter
18	rs75629491	6:31,658,303	Promoter
19	rs1143030	6:31,658,350	Promoter

### Serum concentration of APOM in SLE patients and controls by enzyme-linked immunosorbent assay (ELISA)

The serum levels of APOM in SLE patients and controls were measured by ELISA kit (Yuan Tai Bio Inc., Changsha, China), according to the manufacturer’s instructions. The optical density (OD) of the samples was measured at a wavelength of 450 nm (with a background reading at 620 nm) using an ELX-800 absorbance reader (Bio Tek Instruments, Inc., Winooski, VT, USA). The concentration of APOM in each sample was calculated (as mg/L) from the standard curve.

### Serum levels of anti-dsDNA antibodies in SLE patients and controls by EUROLINE ASSAY

The serum anti-dsDNA antibodiesconcerntrations were detected by using anti-nuclear antibodies (ANA) kit (Euroimmun, Luebeck, Germany) [15]. The Euroline assay is a qualitative in vitro assay containing immunoblot strips coated with parallel highly purified native and recombinant antigens. This assay can detect IgG autoantibodies to 15 antigens: nRNP, Sm, Ro-60, Ro-52, La, Scl-70, PM-Scl, Jo-1, CENP B, PCNA, dsDNA, nucleosomes, histones, ribosomal P-protein, and AMA-M2. The assay was performed according to the recommendations of the manufacturer. The immunoblot strips placed on a work sheet were scanned and evaluated digitally using the EUROline scan. The results were recorded as negative to strong positive based on the numerical value of the signal intensity (SI) displayed.

### Statistical analysis

Continuous data are presented as median (range), and categorical data are expressed as percentages. Continuous data were analyzed with the Student’s t test or one-way analysis of variance (one-way ANOVA) for traits with normal distribution. Variables with a skewed distribution were converted by a logarithmic function before analysis. The Pearson’s correlation analysis was used to test for associations between APOM and variables with a normal distribution. The Spearman correlation analysis was used to analyze variables with skewed distributions. Difference in demographics, variables and genotypes of *APOM* SNP polymorphism variants were evaluated using a Chi^2^ test.

Genotype distributions were tested for Hardy-Weinberg equilibrium using goodness-of-fit test. Allelic, dominant, recessive and additive genetic models were used to test the association between SNP and SLE. Power calculation was performed by Quanto software version 1.2.3 (University of Southern California, Los Angeles, CA, USA). Assuming a minor allele frequency of 0.2486 and disease prevalence of 0.0007%, we had 80% power to detect genetic effects at an OR of 2.34, 3.18 and 5.38 under an additive, dominant, and recessive model in our samples, respectively. Linkage disequilibrium was analyzed by SHEsis software and the result showed complete linkage disequilibriium [16].

All analyses were performed using SPSS 20.0 (IBM, Armonk, NY, USA). Two-sided *P*-values <0.05 were considered statistically significant.

## Results

### Characteristics of the total study population

The clinical parameters of study subjects in the SLE and control groups are described in Table 2. There were no significant differences in gender, age, TC, LDL-C between groups; however, SLE patients demonstrated significant differences in TG, HDL-C, Lpa, hs-CRP, 25 OH-D3, APOA1, APOB, and APOM concentrations (Table 2).

**Table 2:** Patients’ demographics in SLE and controls

Clinical parameter	SLE patients	Healthy Controls	*P* value
Patients (number)	52	52	–
Age (years)	31 (12–52)	32 (20–56)	0.438
Female:Male ratio	46:6	45:7	0.767
Laboratory data			
TG (mmol/L)	1.62 (0.50–8.93)	0.80 (0.35–2.52)	<0.001
TC (mmol/L)	4.18 (1.92–9.28)	4.28 (3.25–5.52)	0.207
HDL (mmol/L)	1.06 (0.35–2.01)	1.44 (1.05–2.15)	<0.001
LDL (mmol/L)	2.50 (0.89–7.06)	2.53 (1.44–3.78)	0.062
APOA1 (g/L)	1.14 (0.57–2.01)	1.45 (1.08–1.89)	<0.001
APOB (g/L)	0.93 (0.38–2.06)	0.89 (0.57–1.25)	0.002
APOM (mg/L)	2.92 (1.54-17.75)	14.80 (3.53–56.07)	<0.001
Lpa (mg/L)	154.15 (9.70–1517.40)	78.80 (3.40–1043.50)	0.041
hs-CRP (mg/L)	2.26 (0.12–125.44)	0.37 (0.04–5.68)	0.008
25-OHD3 (ng/ml)	9.72 (3.00-19.26)	16.16 (6.98-34.22)	<0.001
Anti-dsDNA antibodies (%)	19 (36.5%)	–	–
Anti-SM antibodies (%)	21 (40.4%)	–	–
SLEDAI	15 (5-29)	–	–

Data are presented as median (minimum-maximum). *P* < 0.05 was considered statistically significant

*TG* triglyceride, *TC* total cholesterol, *HDL-C* high-densitylipoprotein cholesterol, *LDL-C* low-density lipoprotein cholesterol, *APOA1* apolipoprotein A1, *APOB* apolipoprotein B, *APOM* apolipoprotein M, *Lpa* lipoprotein(a), *hs-CRP* high-sensitivity C-reactive protein, *SLEDAI* systemic lupus erythematosus disease activity index

### Association between serum APOM levelsand SLEDAI in SLE patients

**Fig. 2:**
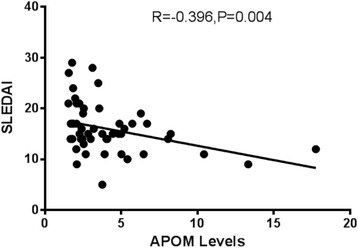
Correlations between APOM levels and SLEDAI, serum APOM levels in patients with SLE were correlated with SLEDAI (*r* = −0.396, *P* = 0.004) in the Pearson analysis

**Fig. 3:**
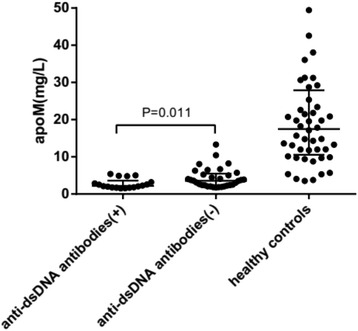
ApoM concentrations in SLE patients and healthy controls, serum APOM levels in SLE paitents with positive anti-dsDNA antibodies were lower than that of patients with negative antibodies (*P* = 0.011)

We tested the serum levels of APOM in SLE patients and controls by ELISA assay. The results showed that the serum levels of APOM was significantly lower in SLE patients than that in healthy controls (Table 2) and correlated with the SLEDAI scores for SLE patients (*r* = −0.396, *P* = 0.004) (Fig. 2). Furthermore, APOM concentrations in SLE patients with positive anti-dsDNA antibodies were dramatically lower than that of patients with negative anti-dsDNA antibodies (*P* = 0.011) (Fig. 3).

### Association between* APOM* SNP and SLE susceptibility

Using DNA sequencing analysis, the frequency of the different genotypes of 19 SNPs located in the promoter region of the *APOM* gene were determined for each individual. Frequencies of the genotypes were determined in patients with SLE (52 individuals) and compared with the frequencies observed in a control group (52 individuals). According to the results of SHEsis soft, we found that rs805264, rs707921 and rs1266078 are complete linkage disequilibrium (Table 3). Nonetheless, no significant association was detected between individual SNPs and SLE (data are not shown).

**Table 3:** Linkage disequilibrium analysis of *APOM* gene rs805264, rs707921 and rs1266078

SNPs	D’	*r* ^2^
rs805264-rs707921	1.000	0.956
rs805264-rs1266078	1.000	0.921
rs707921-rs126078	1.000	1.000

### Analysis of genotypic association of SNP rs805297 with SLE

It has been reported that rs805297 was a risk site for rheumatoid arthritis, therefore, we analyzed the association of rs805297 C/A polymorphism and SLE risk, considering an additive, dominant and recessive genetic model. However, No significant association was detected between SNP rs805297 and SLE (Table 4).

**Table 4:** Analysis of genotypic association of SNP rs805297 with SLE

Gene SNPs	Genotype	Additive model OR (95% CI)	P*	Dominant model OR (95% CI)	*P* ^*&*^	Recessive model OR (95% CI)	*P* ^*#*^
rs805297	CC			1.292 (0.582–2.868)	0.529	2.676 (0.836–8.566)	0.097
	CA	1.011 (0.430–2.377)	0.980				
	AA	2.691 (0.776–9.334)	0.119				

*, &, # Genotype specific *P* values and OR in each additive, dominant or recessive genetic model, respectively, *P* < 0.05 was considered statistically significant

## Discussion

Sphingosine 1-phosphate (S1P), a bioactive lysophospholipid mediator, interacts with vertebrate-specific S1P receptors to regulate various physiological functions and influence immunity in myriad ways. The majority of plasma S1P is complexed with APOM, therefore, it is interesting to explore the immunological functions of the APOM-S1P axis. A study recently has reported that *APOM*−/− mice developed more severe experimental autoimmune encephalomyelitis (EAE), characterized by increased lymphocytes in the central nervous system and breakdown of the blood-brain barrier. APOM-S1P axis was involved in adaptive immune response by restraining bone marrow lymphopoiesis throughinducing S1P/S1P1 receptor signaling in bone marrow progenitors [17]. Because of the important role of APOM in autoimmune disease, it is essential to understand the role of *APOM* genetic variation in relation to autoimmune diseases. Thus, the genetic variation in promoter region of *APOM* which control gene expression can be associated with the disease risk.

The serum concentration of SLE patients was significantly lower than that in controls and correlates with the SLEDAI scores for SLE patients (*r* = −0.396, *P* = 0.004), suggesting that the decreased APOM levels may reflect the severity of disease in SLE patients. It was interesting that anti-dsDNA antibodies were significant biomarkers which reflect the disease activity of SLE, therefore, we suspect that the decrease of APOM in SLE patients may correlate with the elevation of immune complex. Meanwhile, APOM has been characterized as a negative acute response protein in precious researches [18]. The increased hs-CRP levels in SLE patients, which is a marker of systemic inflammation, indicate that the increased inflammatory state caused by rising of immune complex concentration, lead to the decreased levels of APOM. At the same time, we also found that reduction of 25 OH-D3 in SLE patients compared with healthy controls as reported [19]. However, there was no dramatic correlation between 25 OH-D3 and APOM levels. It may be due to the small number of samples. It was reported that Vitamin D had a significant positive relationship with serum HDL and APOA1 [20] and it regulated the expression of APOA1 in HepG2 cells [21]. APOM expression is modulated by several hormonal sinals. So it is likely that vitamin D has effects on the expression of APOM in SLE patients.

According to previous studies, systemic lupus erythematosus carry a high risk of cardiovascular disease (CVD) which is mainly caused by atherosclerosis (AS). The role of APOM on the development and progression of atherosclerosis remain controversial. Although a possible atheroprotective effect of APOM in genetically modified mice, two independent clinical studies found that there were no differences in APOM levels between coronary heart disease (CHD) patients and controls [22]. In present study, the decreased concentrations of APOM may partly caused by the reduction of HDL which was confirmed to be protective in AS.

Lp(a) was thought to be an independent risk factor for the premature development of atherosclerosis. It is involved in different ways: it accumulates in the arterial intima and it activates inflammatory cells and binds to proinflammatory-oxidized-phospholipids [23, 24]. Elevated levels of Lp(a) in patients with SLE, suggesting that it might be involved in the pathogenesis of atherosclerosis. Besides, rising Lp(a) levels merges with acute-phase-protein increase in several diseases such as RA, ischemic stroke, vestibular neuronitis, etc. [25–27]. As the levels of hs-CRP was increased in SLE patients in this study which was consistent with that of Lp(a), it proved that Lp(a) may play an important role in the acute phase cascade reaction process.

To our knowledge, this is the first study to evaluate the role of *APOM *promoter SNPs in relation to SLE patients. *APOM* rs805297 C/A polymorphism was proved to be associated with increased risk of rheumatoid arthritis in Korean and Chinese populations [7, 8]. Although the previous research has reported the association between SNP rs805297 C/A and RA patients, we found that *APOM* rs805297 C/A polymorphism is not a risk factor for genetic susceptibility to SLE in this study. It may be due to the following reasons. At first, the relatively small number of tested individuals in the present study may have masked an increased SLE risk factor due to SNPs in the promoter region of *APOM* (leading to descreases in transcription rates). Alternatively, it is reasonable to expect that *APOM* rs805297 C/A polymorphism may not involve in the regulation of decreased expression of serum APOM protein.

## Conclusions

In this study, functional single-nucleotide polymorphisms (SNPs) in the promoter region of *APOM *did not show a correlation with SLE risk.However, serum APOM levels were decreased in patients with SLE and correlated with disease activity, reflecting APOM could be an indicator of monitoring the progress of systemic lupus erythematosus.

## Additional file

Additional file 1: Figure S1. Study Flowchart. (DOC 1500 kb)

## Abbreviations

APOM: Apolipoprotein M
MALDI-TOF MS: Matrix-assisted laser desorption/ionization time-of-flight mass spectrometry
MHC: Major histocompatibility complex
SLE: Systemic lupus erythematosus
SLEDAI: Systemic lupus erythematosus disease activity index
SNP: Single nucleotide polymorphism

## References

[1] Xiang Y-J, Dai S-M (2009). Prevalence of rheumatic diseases and disability in China. Rheumatol Int.

[2] Feng J-B, Ni J-D, Yao X (2010). Gender and age influence on clinical and laboratory features in Chinese patients with systemic lupus erythematosus: 1790 cases. Rheumatol Int.

[3] Fernando M, Stevens C, Sabeti P, Walsh E, McWhinnie A (2007). Identification of two independent risk factors for lupus within the MHC in United Kingdom families. PLoS Genet.

[4] Rioux J, Goyette P, Vyse T, Hammarström L, Fernando M (2009). International MHC and autoimmunity genetics network mapping of multiple susceptibility variants within the MHC region for 7 immune-mediated diseases. Proc Nat Acad Sci.

[5] Barcellos L, May S, Ramsay P, Quach H, Lane J (2009). High-density SNP screening of the major histocompatibility complex in systemic lupus erythematosus demonstrates strong evidence for independent susceptibility regions. PLoS Genet.

[6] Xu N, Dahlback B (1999). A novel human apolipoprotein(apoM). J Biol Chem.

[7] Huang Y, Liu Y, Jiang L, Sun R, Zhang H (2014). Apolipoprotein m (APOM) levels and APOM rs805297 G/T polymorphism are associated with increased risk of rheumatoid arthritis. Joint Bone Spine.

[8] Hu HJ, Jin EH, Yim SH, Yang SY, Jung SH (2011). Common variants at the promoter region of the APOM confer a risk of rheumatoid arthritis. Exp Mol Med.

[9] Wu X, Niu N, Brismar K (2009). Apolipoprotein M promoter polymorphisms alter promoter activity and confer the susceptibility to the development of type 1 diabetes. Clin Biochem.

[10] Niu N, Zhu X, Liu Y (2007). Single nucleotide polymorphisms in the proximal promoter region of apolipoprotein M gene (apoM) confer the susceptibility to development of type 2 diabetes in Han Chinese. Diabetes Metab Res Rev.

[11] Zhou JW, Tsui SK, Ng MC, Geng H, Li SK (2011). Apolipoprotein M gene (APOM) polymorphism modifies metabolic and disease traits in type 2 diabetes. PLoS One.

[12] Jiao GQ, Yuan ZX, Xue YS (2007). A prospective evaluation of apolipoproteinM gene T-778C polymorphism in relation to coronary artery disease in HanChinese. Clin Biochem.

[13] Zhao D, He Z, Qin X (2011). Association of apolipoprotein M gene polymorphisms with ischemic stroke in a Han Chinese population. J Mol Neurosci.

[14] Tan EM, Cohen AS, Fires JF, Masi AT, McShane DJ (1982). The 1982 revised criteria for the classification of systemic lupus erythematosus. Arthritis Rheum.

[15] Schlumberger W, Meyer W, Proost S, et al. The new EUROBLOT technology: differentiation of auto-antibodies against cell nuclei. Eur J Clin Chem Clin Biochem. 1995;33:116.

[16] Shi YY, He L (2005). SHEsis, a powerful software platform for analyses of linkage disequilibrium, haplotype construction, and genetic association at polymorphism loci. Cell Res.

[17] Galvani BS, Engelbrecht E, Liu C, Steven L, Mari K (2015). HDL-bound sphingosine1-phosphate restrains lymphopoiesis and neuroinflammation. Nature.

[18] Kumaraswamy SB, Linder A, Akesson P, Dahlback B (2012). Decreased plasma concentrations of apolipoprotein M in sepsis and systemic inflammatory response syndromes. Crit Care.

[19] Andreoli L, Dall’Ara F, Piantoni S, Zanola A, Piva N (2015). A 24-month prospective study on the efficacy and safety of two different monthly regimens of vitamin D supplementation in pre-menopausal women with systemic lupus erythematosus. Lupus.

[20] Jorde R, Sneve M, Torjesen P, Figenschau (2010). No improvement in cardiovascular risk factors in overweight and obese subjects after supplementation with vitamin D3 for 1 year. J Intern Med.

[21] Wehmeier K, Beers A, Haas MJ, Wong NCW, Steinmeyer A (2005). Inhibition of apolipoprotein A1 gene expression by 1,25-dihydroxyvitamin D3. Biochim Biophys Acta.

[22] Ahnström J, Axler O, Jauhiainen M, Salomaa V, Havulinna AS (2008). Levels of apolipoprotein M are not associated with the risk of coronary heart disease in two independent casecontrol studies. J Lipid Res.

[23] Kiechl S, Willeit J, Mayr M (2007). Oxidized phospholipids, lipoprotein(a), lipoprotein-associated phospholipase A2 Activity, and 10-year cardiovascular outcomes: prospective results from the bruneck study. Arterioscler Thromb Vasc Biol.

[24] Nordestgaard BG, Chapman MJ, Ray K (2010). Lipoprotein(a) as a cardiovascular risk factor: current status. Eur Heart J.

[25] Dursunoğlu D, Evrengül H, Polat B (2005). lipoprotein p(a) and lipids in patients with rheumatoid arthritis: serum levels and relationship to inflammation. Rheumatol Int.

[26] Wehr H, Rado M, Mendel T, Swiderska M, Bednarska-Makaruk M (2004). Changes in lipoprotein (a)[Lp(a)] level after an ischemic stroke. Neurol Neurochir Pol.

[27] Milionis HJ, Mittari V, Exarchakos G, Kalaitzidis R, Skevas AT (2003). Lipoprotein (a) and acute-phase response in patients with vestibular neuronitis. Eur J Clin Investig.

