# Supplementary figures and images for: Low apolipoprotein M serum levels correlate with Systemic lupus erythematosus disease activity and apolipoprotein M gene polymorphisms with Lupus
Source: Lipids Health Dis. 2017 May 5;16:88. doi: 10.1186/s12944-017-0476-8 (PMC5420091; doi:10.1186/s12944-017-0476-8)

Additional file
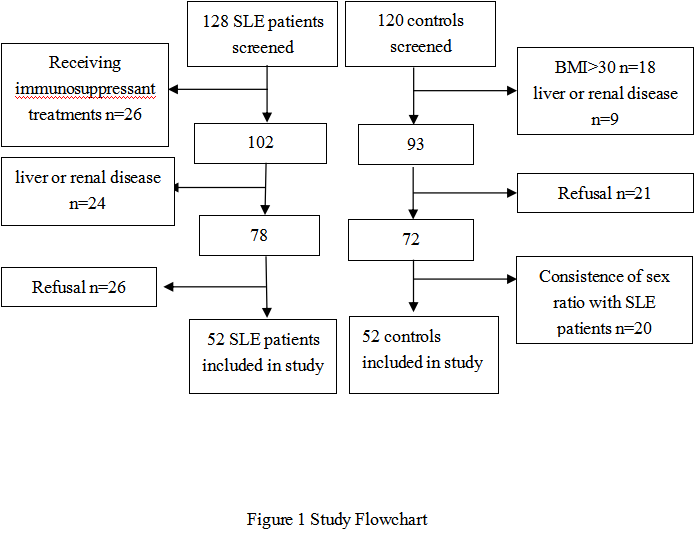
 1

Supplement: Additional file 1: Figure S1. — Study Flowchart. (DOC 1500 kb) [file 12944_2017_476_MOESM1_ESM.doc]
